# Hydrogen Sulfide Signaling Protects *Chlamydomonas reinhardtii* Against Allelopathic Damage From Cyanobacterial Toxin Microcystin-LR

**DOI:** 10.3389/fpls.2020.01105

**Published:** 2020-07-17

**Authors:** Xiao-Dong Chen, Yue Liu, Li-Ming Yang, Xiang-Yang Hu, Ai-Qun Jia

**Affiliations:** ^1^ School of Life and Pharmaceutical Sciences, State Key Laboratory of Marine Resource Utilization in South China Sea, Hainan University, Haikou, China; ^2^ School of Environmental and Biological Engineering, Nanjing University of Science and Technology, Nanjing, China; ^3^ Shanghai Key Laboratory of Bio-Energy Crops, School of Life Sciences, Shanghai University, Shanghai, China; ^4^ College of Biology and the Environment, Nanjing Forestry University, Nanjing, China

**Keywords:** hydrogen sulfide signaling, *Chlamydomonas reinhardtii*, antioxidant enzyme autophagy, proteomic analyses, microcystin-LR

## Abstract

Cyanobacterial blooms have become more frequent and serious in recent years. Not only do massive blooms cause environmental pollution and nutrient eutrophication, but they also produce microcystins (MCs), a group of toxic cycloheptapeptides, which threaten aquatic ecosystem and human health. As such, clarifying the allelopathic interactions between cyanobacteria and other algae is critical to better understand the driving factors of blooms. To date, however, such studies remain largely insufficient. Here, we treated model alga *Chlamydomonas reinhardtii* with microcystin-LR (MC-LR) to determine its allelopathic effects. Results showed that MC-LR markedly suppressed *C. reinhardtii* cell viability. Comparative proteomic and physiological analyses revealed that MC-LR significantly up-regulated protein abundance of antioxidants ascorbate peroxidase (APX) and catalase (CAT) at the beginning stage of exposure. This was accompanied by an over-accumulation of hydrogen peroxide (H_2_O_2_), suggesting that MC-LR suppresses cell viability *via* oxidative damage. Furthermore, we found that MCs induced desulfhydrase (DES) activity for hydrogen sulfide (H_2_S) generation at the beginning stage. Additional H_2_S donors reactivated antioxidant enzyme activity, which reduced H_2_O_2_ accumulation and ultimately enhanced *C. reinhardtii* tolerance to MC-LR damage. This effect could be reserved by inhibiting H_2_S biosynthesis. Simultaneously, we found that H_2_S also suppressed MC-LR-induced cell autophagy, and thus attenuated the toxic effects of MC-LR. Our findings suggest that oxidative bursts may be the main reason for the allelopathic effects of MC-LR on *C. reinhardtii* viability and that H_2_S signaling may enhance *C. reinhardtii* tolerance to MC-LR through the activation of antioxidant enzyme activity and suppression of cell autophagy.

## Introduction

Toxic cyanobacterial algal blooms have become more frequent and serious in freshwater environments in recent years due to the effects of climatic change and nutrient overload. These blooms threaten ecosystem functioning and are directly harmful to aquatic algae, macrophytes, invertebrates, fish, and humans (Duan et al., 2009; Paerl and Paul, 2012; Jiang et al., 2014). Among the large cyanobacterial family, *Microcystis aeruginosa* is the most common bloom-forming species due to its wide distribution, which has reached up to 70% of the global cyanobacteria biomass, and has strong ability to produce microcystin (MC) toxins. MCs belong to a group of cycloheptapeptide inhibitors of intracellular protein phosphatases 1&2A (PP1/PP2A). These toxins have been found to have adverse effects on the liver, small intestine, colon, brain, kidney, lung, heart, and reproductive system, and also exhibit potential cancer-promoting activity (Mankiewicz-Boczek et al., 2006; Chen et al., 2009; Xu et al., 2018). In recent years, the toxic effects of MCs have been well documented in aquatic microorganisms, phytoplankton, zooplankton, and fish (Hitzfeld et al., 2000; Weng et al., 2007; Mezhoud et al., 2008). To date, however, the related mechanism of MC toxicity remains poorly known.

MCs are reported to damage animal tissue by generating reactive oxygen species (ROS) and triggering oxidative stress (Shi et al., 2017). Oxidative stress occurs after ROS over-accumulation, which can be overcome by the cellular antioxidant enzymes, such as APX or CAT (Wang et al., 2019; Liu Y. et al., 2020; Zhao et al., 2020). For example, MCs extracted from the cyanobacterium *Radiocystis fernandoi* strain R28 have been shown to induce anemia and oxidative stress in fish erythrocytes (Sakuragui et al., 2019). In plants, small molecular antioxidants, such as glutathione and ascorbate acid, can also efficiently scavenge ROS accumulation (Vaidyanathan et al., 2003). Hydrogen sulfide (H_2_S), a small gaseous molecule, is involved in multiple physiological functions in mammals, such as blood vessel relaxation, neurotransmission, insulin signaling, and angiogenesis (Wang, 2012; Li et al., 2016). H_2_S is also reported to enhance plant tolerance to environmental stress (Lisjak et al., 2013). In plants, cysteine desulfhydrases, including L-cysteine and D-cysteine desulfhydrases, catalyze the degradation of cysteine into H_2_S, ammonia, and pyruvate (Papenbrock et al., 2007). Desulfhydrases (DES) are members of the *O*-acetyl-L-serine(thiol)lyase protein family and are mainly responsible for the generation of H_2_S in *Arabidopsis* (Li et al., 2016; Mei et al., 2019). Previous studies have demonstrated that H_2_S signaling enhances plant tolerance to environmental or metal stress by up-regulating antioxidant enzyme activity to reduce ROS over-accumulation (Alvarez et al., 2012; Corpas et al., 2019). H_2_S is also reported to negatively regulate cell autophagy and increase the accumulation of ATG8, which is an important marker of autophagy (Gonzalez-Ballester et al., 2010; Alvarez et al., 2012; Gotor et al., 2013). Our previous study further showed that nitric oxide (NO), another small molecule gas, can enhance *C. reinhardtii* tolerance to salt stress by modulating cell autophagy (Chen et al., 2016). It is reported that ROS can trigger cell autophagy in algae after MC treatment (Liu et al., 2018), suggesting the possible role of H_2_S signaling and cell autophagy in MC-induced damage or the allelopathic interaction of cyanobacteria and other algae. However, relevant evidence has not yet been reported.

To better understand the allelopathic interactions between cyanobacteria and other algae, we treated unicellular *C. reinhardtii* with the cyanobacterial toxin MC-LR. Using comparative proteomics and physiological experiments, we found that MC-LR induced the differential expression of several proteins, including APX and CAT, induced the over-accumulation of ROS, mainly hydrogen peroxide (H_2_O_2_), and increased the activities of APX and CAT at the early stage of exposure. We also found that MC-LR induced DES activity for the release of H_2_S. Interestingly, additional H_2_S also enhanced the activity of APX and CAT to efficiently scavenge ROS production, and thus ultimately improved the tolerance of *C. reinhardtii* to MC-LR. H_2_S signaling also suppressed MC-LR-induced cell autophagy and thus reduced toxic damage in *C. reinhardtii.* Our findings revealed a novel mechanism by which H_2_S is involved in the allelopathic interaction between cyanobacteria and other algae.

## Materials and Methods

### Growth of *C. reinhardtii* and MC-LR Treatment

The *C. reinhardtii* wild-type strain was purchased from the Freshwater Algae Culture Collection Center at the Institute of Hydrobiology (Chinese Academy of Sciences (CAS), Wuhan, China). Cells were cultured under continuous light at 23°C in Tris-acetate-phosphate (TAP) medium. MC-LR was purchased from the Sigma Chemical Company (St. Louis, MO, USA). For MC-LR treatment, *C. reinhardtii* cells in the stationary phase (10^6^ cells/ml) were treated with different concentrations of MC-LR. At different time points, cells were removed for further analysis.

### H_2_S Signal Donors and Inhibitor Treatments

The H_2_S artifical donors NAHS or GYY, and H_2_S scavenger hypotaturine (HY) was used for investigating H_2_S function as previous report (Garciamata and Lamattina, 2010). Aminooxyacetic acid was used as special inhibitor to suppress the activity of cystathionine-beta-synthase (CBS)/cystathionine-gamma-lyase (CSE), which is responsible for H_2_S biosynthesis in organism (Asimakopoulou et al., 2013; Ahmad and Szabo, 2016). For chemical treatments, the H_2_S artificial donor NAHS or GYY, H_2_S scavenger HY, H_2_S biosynthesis enzyme inhibitor AOA was used, respectively. These chemicals were added into the alga liquid culture containing MC-LR, the sample was collected at indicated time for further analysis.

### Viability Assay

Evans blue dye E2129 (Sigma, St. Louis, MO, USA) was used as a marker of cell death (Voigt et al., 2017). In brief, *C. reinhardtii* cells (45 ml, 10^6^ cells/ml) were incubated with 0.1% (w/v) Evans blue for 5 min, then washed once with 50 ml of TAP medium, and re-suspended in an equal volume of TAP medium. Viability was determined by counting the colored cells in a hemocytometer, with 10 random counts performed. Control tests of viability were performed with suspension cultures fixed in 70% ethanol or in FAA (5% formaldehyde, 5% glacial acetic acid, 70% ethanol, by vol.). The photosynthesis Fv/Fm ratio was measured as reported (Chen et al., 2016).

### Total Protein Extract for iTRAQ Analysis

Total proteins were extracted from cultured algal cell samples using phenol extraction (Cheng et al., 2016). Briefly, after MC-LR treatment, 50 ml of algal culture was centrifuged at 5,000*g* for 15 min at 4°C. The resulting pellet was collected, frozen in liquid N_2_, and well ground using a mortar and pestle, keeping the sample submerged in liquid N_2_. Ice-cold protein extraction buffer (10 ml) containing KCl (100 mM), Triton X-100 (1% v/v), β-mercaptoethanol (1% v/v), L-ascorbic acid (50 mM), and phenylmethanesulfonyl fluoride (1 mM) in Tris-HCl buffer (100 mM, pH 7.8) was used and powdered, and the suspension was mixed with an equal volume of Tris-phenol (pH 8.0) and vortexed thoroughly. The upper phenolic phase was obtained after centrifugation at 12,000*g* for 30 min at 4°C and mixed with five volumes of ammonium acetate (10 mM) in methanol, with the mixture kept at −20°C overnight. The protein pellet was then obtained after centrifugation at 12,000*g* for 15 min at 4°C, followed by washing in cold acetone containing 0.1% β-mercaptoethanol and drying for storage at −80°C. To dissolve the protein pellet, Tris–HCl lysis buffer (pH 8.5, 40 mM) containing urea (7 M), EDTA (2 mM), thiourea (2 M), CHAPS (4% v/v), and PMSF (1 mM) was added at a final ratio of 10 mg/ml, with the solution sonicated at 200 W for 15 min to accelerate dissolution, then centrifuged for 15 min at 12,000*g* in a cold room. The pellet was discarded, and the supernatant was transferred to another tube. After this, 10 mM DTT was added to reduce the disulfide bond, iodoacetamide (IAM, 55 mM) was added under dark conditions to covalently block cysteine, and five volumes of cold acetone were added under −20°C for 2 h to the pellet protein, which was then dried and re-dissolved in 500 μl TEAB (tetraethylammonium bromide, 0.5 M) and centrifuged for 15 min at 12,000*g* in a cold room. The protein concentration was quantified by the Bradford method (Whiffen et al., 2007) using a Bio-Rad protein assay kit (Bio-Rad, Hercules, CA, USA).

### iTRAQ Mass Spectrometry

Extracted protein (100 µg) from *C. reinhardtii* was used for iTRAQ analysis. We first digested the protein using Gold Trypsin (Promega, Madison, WI, USA) at a ratio of 30:1 (protein/trypsin) for 16 h at room temperature. The digested peptides were dried using vacuum centrifugation, and then dissolved in TEAB buffer (0.5 M) as recommended by the protocols for the 8-plex iTRAQ reagent (AB Sciex Inc., MA, USA). One unit of iTRAQ reagent 113 to 117 isobaric tags in 24 μl of isopropanol was used to label the sample for 2 h under room temperature. After labeling, the peptide mixture was pooled and dried using vacuum centrifugation, then re-suspended in 4 ml of dissolution buffer containing 25 mM NaH_2_PO_4_ and 25% acetonitrile (pH 2.7). The sample was separated by an Ultremex strong cationic exchange column (4.6 × 250 mm) in a Shimadzu LC-20AB HPLC Pump system (Kyoto, Japan). Sample fractions were eluted with a linear gradient flow at 1 ml min^−1^ and an elution program of 5% buffer B (1 M KCl and 25 mM NaH_2_PO_4_ in 25% ACN, pH 2.7) for 7 min, 5–60% gradient buffer B for 20 min, 60–100% buffer B for 2 min, and maintenance with 100% buffer B. The elution was divided into 20 fractions by monitoring absorbance at 214 nm, and then desalted using a C-18 column. The desalted fraction was dissolved in buffer C containing 5% ACN and 0.1% formic acid at a ratio of 0.5 µg µl^−1^ and centrifuged at 20,000*g* for 10 min at 4°C. For high-performance liquid chromatography (HPLC)-mass spectrometry (LC-MS-MS), 5 μl of the supernatant was loaded by the sampler onto a Shimadzu LC-20 AD nanoHPLC (Kyoto, Japan) with a C18 column (200 µm inner diameter). The peptide was eluted with a linear gradient program, with a washing program of 5% buffer D (95% ACN, 0.1% formic acid) for 5 min, 3–35% buffer D for 35 min, 60–80% buffer D for 2min, and maintenance in 80% buffer D. Mass spectrometry following HPLC was performed using a TripleTOF 5600 system (AB SCIEX, Concord, ON, Canada) equipped with a Nanospray III source, and raw data were obtained under ion spray voltage at 2.5 kV, 30 psi N gas, 15 psi nebulize gas, and heater temperature at 150°C. The instrument was operated in information dependent acquisition (IDA) mode, with mass range between 100–2 400 m/z and detection resolution over 30,000 (FWHM) in the Orbitrap analyzer set for TOF-MS scans. The 30 most intense ions above a threshold of 120 cps with a 2^+^ to 5^+^ charge state were selected for fragmentation. A sweeping collision energy at 35 ± 5 eV coupled with iTRAQ adjusted rolling collision energy was used for all precursor ions during collision-induced dissociation.

### Bioinformatics Analysis

The raw MS/MS data files were converted to Mascot generic files by Proteome Discoverer 1.2 and searching was performed using the *C. reinhardtii* proteome database (http://plants.ensembl.org). Peptide mass and fragment mass tolerance values were 10 ppm and 0.1 Da, respectively, and one missing cleavage in the trypsin digestion was allowed. For all searching, oxidation of methionine residues and pyroglutamate formation of N-terminal glutamine residues were set as the variable modifications, and carbamidomethyl formation of cysteine residues and iTRAQ labeling of lysine sites were set as the fixed modifications. Only peptides with significance scores over 20 at a 99% confidence interval and false discovery rate (FDR) of less than 1.5% were selected for quantification. Cutoffs of 1.5- or 0.6-fold with a *p*-value of less than 0.05 were set to indicate significantly differential protein expression.

### *In Situ* H_2_O_2_ Staining and Content Analysis

For *in situ* detection of H_2_O_2_, 3, 3’-diaminobenzidine (DAB) staining was performed as described previously (Chen et al., 2016). In brief, the cell culture was treated with MC-LR for indicated time, collected by centrifugation at 3,000*g* for 10 min at 22°C, and then mixed with 1 mg ml^−1^DAB containing 0.05% v/v Tween-20 and 10 mM sodium phosphate buffer (pH 7.0). The reaction was terminated at 6–7 h post-inoculation when a brown precipitate became visible in the cell after microscope observation. The cells were then fixed for 15 min in ethanol:acetic acid:glycerol (3:1:1) and destained with acetone for 12 h. The cells were observed by light microscopy under bright field at 100× magnification. The H_2_O_2_ levels were determined using a Synergy 2 Multi-mode Reader (Biotek, Winooski, VT, USA) equipped for excitation in the range of 530–560 nm and fluorescence emission at 590 nm. The H_2_O_2_ concentration was calculated using a H_2_O_2_ standard curve.

### Antioxidant Enzyme Activity Assay

To determine antioxidant enzyme activities, 10 ml of algal culture was centrifuged at 3,000*g* for 15 min at 4°C. The resulting pellet was quickly homogenized in liquid nitrogen and extracted with 5 ml of extraction buffer [50 mM sodium phosphate buffer (pH 7.0), 0.2 mM EDTA, and 2% polyvinylpolypyrrolidone (PVPP)] for 10 min. The homogenates were filtered through two layers of cheesecloth and centrifuged at 4°C and 15,000*g* for 15 min. The supernatants were desalted on a Sephadex G-50 column and used to measure enzymatic antioxidant activities. Total soluble protein concentration of the supernatants was determined by the Bradford method. The activities of APX and CAT were determined as described previously (Bernstein et al., 2010; Chen et al., 2016).

The activity of APX was determined based on the oxidation of ascorbate. The reaction solution (3 ml) contained 0.1mM sodium acetate buffer (pH 5.4), 1 M EDTA, 0.8 mM H_2_O_2_, 0.17 mM ascorbate, and 100 μl of extracted solution. The reaction was initiated by adding the enzyme extract. Changes in absorbance at 290 nm were read every 10 s for 60 s using a spectrophotometer (Spectronic Genesys Series; Spectronic Instruments, Rochester, NY, USA). Enzyme activity was quantified using the molar extinction coefficient for ascorbate (2.8 mM^− 1^ cm^−1^) and results were expressed in μmol min^−1^ gFW^−1^ (gFW, gram fresh weight), taking into consideration that 2 mol of ascorbate is required for reduction of 1 mol of H_2_O_2_. Results from four independent replicates were analyzed (usually with two subsampled plants per replicate).

The activity of CAT was determined based on the oxidation of H_2_O_2_. The reaction solution (1.5 ml) contained 35 mM phosphate buffer (pH 7.0), 15 mM H_2_O_2_, and 100 μl of extracted solution. The reaction was initiated by adding the enzyme solution. Changes in absorbance at 240 nm were read every 10 s for 90 s using a spectrophotometer. Enzyme activity was quantified using the molar extinction coefficient for H_2_O_2_ (36 M^−1^ cm^−1^) and results were expressed in μmol min^−1^ gFW^− 1^. Results from three to eight independent replicates were analyzed (usually with two subsampled plants per each replicate).

### H_2_S Content and DES Enzyme Activity Analysis

After MC-LR treatment, the cells (50 ml) were harvested and ground to a fine powder with liquid nitrogen and extracted in 10 ml of phosphate-buffered saline (PBS, pH 6.8, 50 mM) containing 0.1 mM EDTA and 0.2 mM ascorbic acid. The homogenate was mixed in a test tube containing 100 mM PBS (pH 7.4), 10 mM L-Cys, and 2 mM phosphopyridoxal at room temperature, and the released H_2_S was absorbed in a zinc acetate trap. The trap consisted of a small glass tube containing 3 ml of 0.5% (w/v) zinc acetate fixed to the bottom of the reaction bottle. After 30-min reaction, 0.3 ml of 20 mM dimethyl-*p*-phenylenediamine was dissolved in 7.2 mM HCl and added to the trap. This was followed by injection of 0.3 ml of 30 mM ferric ammonium sulfate in 1.2 ml of HCl. After incubation for 15 min at room temperature, the amount of H_2_S in the zinc acetate trap was determined colorimetrically at 667 nm. A calibration curve was established by NaHS according to the above method, and H_2_S content in *C. reinhardtii* was expressed as nmol g^−1^ fresh weight.

DES activity was measured by the release of sulfide from L-Cys, as described previously (Ma et al., 2015). The assay contained a total volume of 1 ml: 1 mM dithiothreitol, 1 mM L-Cys, 100 mM Tris–HCl, pH 8.0, and enzyme extract. The reaction was initiated by the addition of L-Cys. After incubation for 15 min at 37°C, the reaction was terminated with the addition of 100 ml of 30 mM FeCl_3_ dissolved in 1.2 N HCl and 100 ml of 20 mM N,N-dimethyl-p-phenylenediamine dihydrochloride dissolved in 7.2 N HCl. The formation of methylene blue was determined at 670 nm, and enzyme activity was calculated using the extinction coefficient of 15 × 10^6^ cm^2^ mol^−1^ (Ma et al., 2015).

### RNA Extraction and Quantitative Real-Time Polymerase Chain Reaction (qRT-PCR)

For qRT-PCR analysis, *C. reinhardtii* samples were collected after different treatments to extract total RNA using the Trizol reagent (Invitrogen, Carlsbad, CA, USA). First-strand cDNA synthesis and qRT-PCR analysis were performed as described previously (Liu et al., 2020). The primers used for qRT-PCR are listed in **Supplemental Table 1**. For each sample, the experiment was repeated three times.

### Immunoblot Analysis

Immunoblotting was performed as described previously (Cheng et al., 2016). Total protein extracts were separated on 15% SDS-PAGE gel. Equal amounts of sample were loaded into the wells. For immunoblot analysis, the protein samples were electroblotted onto polyvinylidene difluoride (PVDF) membranes using a Trans-Blot well (Bio-Rad, Hercules, CA, USA). After transfer, the membranes were probed with the appropriate primary antibodies and horseradish peroxidase (HRP)-conjugated goat secondary antibody (Promega, Madison, WI, USA), and signals were detected using an ECL Kit (GE Company, Evansville, IN, USA). The primary antibodies against *C. reinhardtii* tubulin and ATG8 (Cat. AS142769) were obtained from AgriSera (Vännäs, Sweden), and diluted 1:3,000 for anti-tubulin and 1:1,000 for ATG8.

## Results

### Effects of Cyanobacterial Toxin MC-LR on *C. reinhardtii* Growth

**Figure 1:**
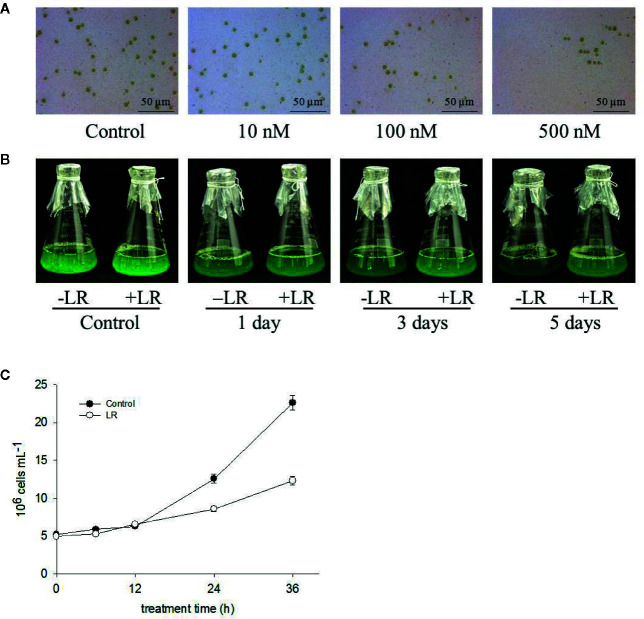
MC-LR suppresses growth of *C. reinhardtii.* **(A)** Dose effect of MC-LR on growth of *C. reinhardtii.* MC-LR at different concentrations was used to treat 3-d-old *C. reinhardtii* cells for 7 d. Cell phenotype was then photographed. **(B)** Time-course effect of 100 nM MC-LR on growth of *C. reinhardtii.* MC-LR (100 nM) was used to treat 3-d-old *C. reinhardtii* cells for various periods of time, with images then taken. Culture without MC-LR treatment was used as the control. **(C)** Cell density of *C. reinhardtii* after 100 nM MC-LR treatment. Here, 3-d-old *C. reinhardtii* cells were treated with 100 nM MC-LR for indicated days, with cell density then calculated at indicated days. Values are means ± SD of three biological replicates.

To determine the toxic effects of MC-LR on the growth of *C. reinhardtii*, we first examined the dose effect of MC-LR on *C. reinhardtii* biomass. The *C. reinhardtii* cells were cultured in TAP medium for 3 d, followed by treatment with different concentrations of MC-LR for 7 d. After this, we checked cell viability and biomass. As shown in **Supplemental Figure 1**, at concentrations below 10 nM, MC-LR slightly repressed the growth of *C. reinhardtii*, accompanied by low cell death, as determined by Evans blue staining. Once the concentration of MC-LR was over 100 nM, cell biomass was significantly reduced by 40% and cell death dramatically increased, reaching over 80% at 300 nM MC-LR. Thus, we selected 100 nM MC-LR for further study. As shown in **Figures 1A, B**, cell chlorophyll content after 100nM MC-LR treatment was markedly lower than that of the control without MC-LR treatment (**Supplemental Figure 2**), indicating that MC-LR suppressed cell growth. Cell density was also reduced after MC-LR treatment (**Figure 1C**). Thus, these results suggest that MC-LR treatment suppresses cell viability and increases cell damage of *C. reinhardtii.*

### iTRAQ Analysis of Dynamic Protein Profiles in *C. reinhardtii* After MC-LR Treatment

**Figure 2:**
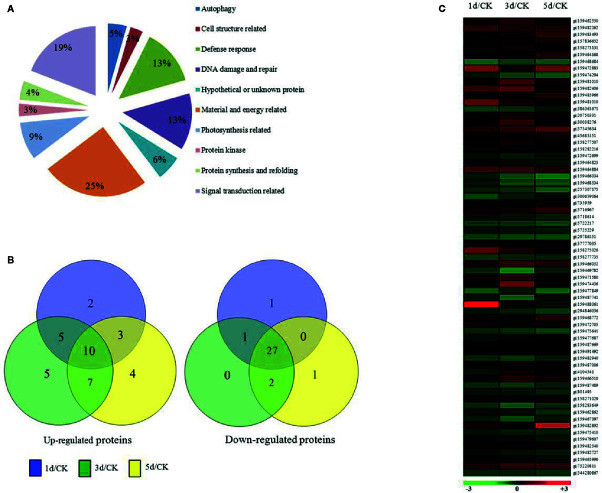
Expression profile of *C. reinhardtii* protein species affected by exposure to MC-LR for various periods of time. **(A)** Functional classification, **(B)** Venn diagram analysis, **(C)** and hierarchical clustering of protein species expression profiles based on samples obtained after indicated periods of MC-LR treatment. Analyses were repeated three times with similar results. Hierarchical cluster analysis was conducted using Cluster 3.0 and Treeview (http://bonsai.ims.u-tokyo.ac.jp/~mdehoon/software/cluster).

To understand the mechanism of the *C. reinhardtii* response to MC-LR, we performed comparative iTRAQ proteomics to monitor the dynamic protein changes after 100 nM MC-LR treatment. Samples were collected at 1, 3, and 5 d for iTRAQ labeling. Samples without MC-LR exposure were used as the control. Each iTRAQ experiment was replicated three times. Protein prediction after data collection was conducted using Mascot software and the public *C. reinhardtii* protein database. Protein abundance was divided into three groups: group 1 (1 d of MC-LR treatment/untreated control), group 2 (3 d of MC-LR treatment/untreated control), and group 3 (5 d of MC-LR treatment/untreated control), with a fold-change cut off of >1.5-fold for significantly increased expression and <0.6-fold for significantly decreased expression. In total, we detected 68 proteins with a significant change in expression in response to MC-LR treatment (*p <*0.05) (**Table 1**). These proteins were divided into nine groups based on their biological function; most were classified by material and energy metabolism, followed by plant antioxidant proteins, DNA damage and repair, autophagy-related, photosynthesis, signal transduction, and defense related proteins (**Figure 2A**). Among the differentially expressed proteins, 20 were up-regulated and 29 were down-regulated after 1 d of treatment, 27 were up-regulated and 30 were down-regulated after 3 d of treatment, and 24 were up-regulated and 30 were down-regulated after 5 d of treatment (**Figure 2B**).

**Table 1:** MC-LR induced different proteins in *Chlamydomonas* after 100nM MC-LR stress for 5d identified by iTRAQ.

NCBI Accession No.^a^	Protein Name	Ratio					
		1d/CK	p value	3d/CK	p value	5d/CK	p value
Autophagy							
gi|159462550	autophagy protein VPS30	1.52	0.009	1.82	0.012	1.57	0.022
gi|159482262	autophagy protein 8	1.89	0.011	1.75	0.041	1.80	0.046
gi|159483493	autophagy protein 3	1.12	0.017	1.56	0.035	1.93	0.015
Cell structure related							
gi|157836652	Chain F	0.89	0.006	1.42	0.043	1.67	0.021
gi|159482014	actin	1.44	0.018	1.60	0.025	1.32	0.029
Defense response							
gi|159464168	Thioredoxin M-type	1.13	0.006	1.21	0.04	1.86	0.005
gi|159468684	chaperonin 60B2	0.12	0.021	0.26	0.024	0.17	0.020
gi|159472883	thioredoxin h1	2.27	0.012	1.19	0.017	2.33	0.042
gi|159474294	heat shock protein 90A	0.50	0.012	0.23	0.007	0.15	0.042
gi|159488379	L-ascorbate peroxidase	1.38	0.023	2.02	0.016	1.61	0.024
gi|159482406	glutathione-S-transferase	1.94	0.010	2.13	0.011	1.53	0.040
gi|159485966	Heat shock 22 kDa protein, chloroplastic	1.11	0.029	1.51	0.028	1.83	0.031
gi|2388691	catalase	2.17	0.016	1.65	0.024	1.50	0.019
gi|386363671	mitochondrial chaperonin 60 precursor	0.27	0.010	0.32	0.028	0.53	0.009
DNA damage and repair							
gi|20750301	serine acetyl transferase	1.02	0.009	1.65	0.047	1.39	0.019
gi|30038276	REX1-B	1.38	0.015	1.97	0.025	1.33	0.033
gi|37545634	RNA-binding protein RB38	1.69	0.033	1.90	0.022	2.19	0.031
gi|45685351	putative DNA repair protein	0.94	0.019	1.14	0.044	1.69	0.043
gi|159470203	DNA repair protein	1.40	0.020	1.65	0.035	1.38	0.044
gi| 159480812	DNA damage inducible protein	0.76	0.020	1.24	0.007	1.07	0.021
gi|159472699	RAN binding protein, RANBP1, partial	0.58	0.022	0.43	0.006	0.56	0.050
gi|159464823	DEAH-box helicase, possible nuclear pre-mRNA splicing factor	0.75	0.046	0.62	0.044	0.55	0.004
gi|159464884	histone H3	1.96	0.049	1.99	0.026	1.86	0.018
Material and energy related							
gi|735959	aconitate hydratase	0.97	0.012	0.84	0.015	1.56	0.016
gi|5716967	ADP-ribosylation factor-like protein 13B.	1.52	0.030	1.66	0.002	1.96	0.004
gi|5718614	Adenylosuccinate synthetase	0.62	0.012	0.53	0.023	0.42	0.016
gi|5722217	S-adenosylmethionine synthase	0.32	0.008	0.30	0.001	0.23	0.026
gi|159483707	Glutamine synthetase cytosolic isozyme	0.56	0.040	0.52	0.002	0.49	0.048
gi|29786351	ATP syntase-associated protein ASA1	0.26	0.016	0.20	0.036	0.19	0.032
gi|37777035	Matrix metalloprotease	1.57	0.021	1.16	0.011	1.22	0.039
gi| 6573214	nitrate reductase	2.16	0.035	1.74	0.047	1.51	0.038
gi| 159469564	adenylosuccinate synthase	0.30	0.014	0.37	0.039	0.40	0.013
gi|159466052	low-CO2 inducible protein	1.56	0.002	2.00	0.004	1.90	0.034
gi|159469782	glutamine synthetase	0.39	0.012	0.12	0.001	0.44	0.017
gi| 5726568	D-Cys desulfhydrase	1.54	0.043	1.87	0.027	1.34	0.045
gi| 5726998	OASTL2/cysteine synthase	1.13	0.031	2.28	0.006	0.93	0.025
gi|159477849	ubiquinol: cytochrome c oxidoreductase 5	0.17	0.009	0.49	0.041	0.12	0.043
gi|159487741	Transketolase	0.54	0.024	0.13	0.043	0.58	0.045
gi|5721691	OASTL1/cysteine synthase	3.43	0.032	0.82	0.032	0.57	0.015
gi|294846036	LEU1Sm	0.52	0.005	0.4	0.035	0.26	0.032
Photosynthesis related							
gi|159468772	light-harvesting protein of photosystem I	1.53	0.011	1.14	0.037	1.91	0.040
gi|159472705	ribosomal protein S1 homologue	1.51	0.002	0.82	0.025	1.57	0.022
gi|159475641	minor chlorophyll a-b binding protein of photosystem II	0.38	0.041	0.31	0.049	0.23	0.013
gi|159477687	prohibitin	0.61	0.020	0.78	0.036	0.88	0.040
gi|159487669	chloroplast elongation factor G	1.27	0.009	1.37	0.003	1.33	0.032
gi|159491492	light-harvesting complex II chlorophyll a-b binding protein M3	0.93	0.047	1.39	0.005	1.27	0.003
Protein kinase							
gi|159482940	phosphoglycerate kinase	0.38	0.029	0.28	0.011	0.45	0.001
gi|159487006	galactose kinase	1.29	0.018	1.43	0.028	1.33	0.029
Protein synthesis and refolding							
gi|4104541	protein disulfide isomerase	0.57	0.010	1.63	0.017	1.35	0.026
gi|159466510	eukaryotic initiation factor 4A-like protein	1.42	0.032	1.80	0.016	1.68	0.011
gi|159487489	protein disulfide isomerase	0.36	0.025	0.27	0.047	0.42	0.002
Signal transduction related							
gi|801495	Cytochrome c oxidase subunit 1	0.54	0.043	0.52	0.031	0.41	0.007
gi|159477028	14-3-3 protein	0.98	0.049	1.21	0.029	1.30	0.036
gi|5718946	calreticulin 2, calcium-binding protein	0.35	0.022	0.14	0.016	0.34	0.046
gi|159462862	Calreticulin 2	0.62	0.049	0.55	0.03	0.32	0.046
gi|159467397	ran-like small GTPase	0.47	0.035	0.20	0.008	0.50	0.036
gi|159482892	Caltractin	1.59	0.038	1.18	0.044	2.68	0.040
gi|159475410	HISTONE DEACETYLASE 9	0.52	0.023	0.43	0.017	0.41	0.012
gi|159479607	translation initiation factor eIF-2B subunit beta	0.78	0.045	0.65	0.003	0.53	0.043
gi|159482540	DnaJ-like protein and Myb-like transcription factor	0.96	0.006	1.23	0.004	1.52	0.037
gi|159482727	General transcription factor IIH subunit	0.63	0.020	0.52	0.036	0.46	0.042
gi|159465996	bZIP transcription factor	0.85	0.001	0.75	0.026	0.63	0.042
gi|75220811	Nitrate transporter 2.1	1.68	0.007	1.96	0.013	1.99	0.011
gi|544280867	Axonemal coiled-coil protein ODA10	0.43	0.036	0.41	0.024	0.36	0.035
Hypothetical or unknown protein							
gi|159466034	hypothetical translation initiation factor	0.48	0.011	0.19	0.031	0.04	0.005
gi|159468534	predicted protein	0.50	0.043	0.21	0.008	0.18	0.015
gi|257307175	unnamed protein product	0.42	0.003	0.32	0.009	0.13	0.017
gi|300659064	unnamed protein product	0.23	0.023	0.47	0.048	0.65	0.041

We also conducted hierarchical cluster analysis to categorize the proteins that showed differential expression profiling during MC-LR toxicity stress (**Figure 2C**). The abundance of antioxidant proteins, such as L-ascorbate peroxidase (APX, gi|159488379), glutathione-S-transferase (gi|159482406), and catalase (CAT, gi|2388691), were up-regulated. Several proteins associated with autophagy, such as autophagy protein VPS30 (gi|159462550), autophagy protein 8 (gi|159482262), and autophagy protein 3 (gi|159483493), and other proteins, such as the bZIP transcription factor (gi|159465996), DnaJ-like protein and Myb-like transcription factor (gi|159482540), signal transduction related protein 14-3-3 protein (gi|159477028), and refolding related protein disulfide isomerase (gi|4104541), were also differentially regulated after MC-LR treatment, suggesting that MC-LR damaged *C. reinhardtii* cell viability through different strategies.

### MC-LR Induced Generation of ROS and Impaired Antioxidant Enzyme Activities

**Figure 3:**
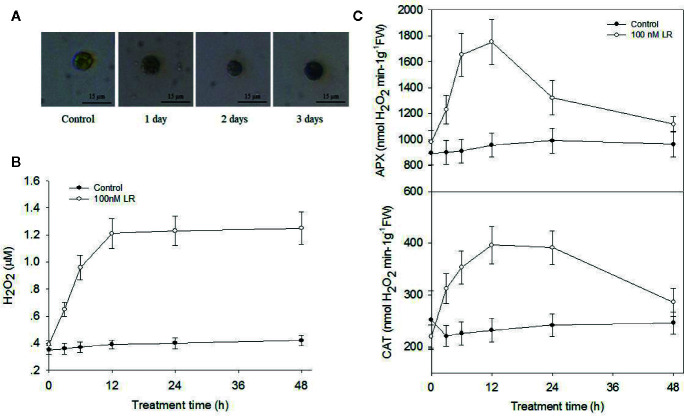
MC-LR induced production of H_2_O_2_ and increased antioxidant APX and CAT activities. **(A)** *In-situ* detection of H_2_O_2_ production by DAB staining. Here, 3-d-old *C. reinhardtii* cells were treated with 100 nM MC-LR for indicated days, and H_2_O_2_ accumulation was then observed by DAB staining. **(B)** Quantification of H_2_O_2_ production in *C. reinhardtii* cells by MC-LR treatment. Here, 3-d-old *C. reinhardtii* cells were treated with 100 nM MC-LR for indicated days, and H_2_O_2_ accumulation was then quantified. Cells without MC-LR treatment were used as the control. Values are means ± SD of three biological replicates. **(C)** Quantification of the antioxidant APX and CAT enzyme activities in *C.reinhardtii* cells by MC-LR treatment. Here, 3-d-old *C.reinhardtii* cells were treated with 100 nM MC-LR for indicated days, with enzyme activity of APX and CAT then measured. Cells without MC-LR treatment were used as the control. Values are means ± SD of three biological replicates.

Our above proteomics data showed that MC-LR treatment increased protein abundance of a series of antioxidant enzymes, including APX and CAT. Our previous study also showed that environmental stress, e.g., salinity, induced a rapid increase in ROS, mainly H_2_O_2_, and damage to cell viability (Chen et al., 2016). Therefore, it is possible that low concentrations of MC-LR also trigger protective antioxidant enzyme activity for the scavenging of H_2_O_2_ damage. To test this, we first determined the accumulation of H_2_O_2_ by *in-situ* DAB staining. A strong brown color was observed in the algal cells after 12 and 24 h of MC-LR treatment (**Figure 3A**). In agreement, quantification of H_2_O_2_ generation in the cell culture showed that MC-LR treatment dramatically increased H_2_O_2_ production (**Figure 3B**), indicating high accumulation of H_2_O_2_. Our proteomics data also showed that MC-LR treatment increased protein abundance of antioxidant-associated enzymes, such as APX and CAT, hinting at their protective function against ROS damage. We then measured the antioxidant enzyme activities of APX and CAT after MC-LR treatment. MC-LR treatment indeed induced an increase in APX and CAT activity during the first 2 d of MC-LR treatment, followed by a gradual reduction after 3 d of MC-LR treatment (**Figure 3C**). These results suggest the possible protective function of these antioxidants in algae during the early stage of MC-LR exposure.

### MC-LR Induced Accumulation of H_2_S and DES Enzyme Activity

**Figure 4:**
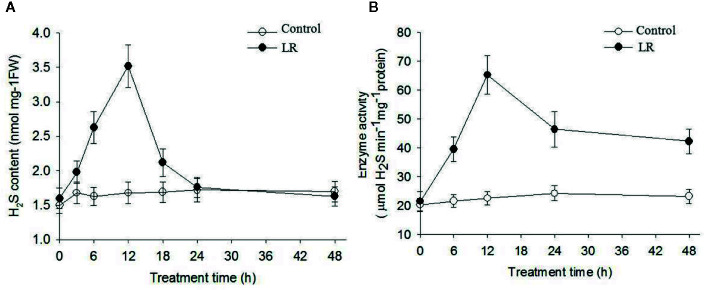
MC-LR induced production of H_2_S and increased enzyme activity of DES. Here, 3-d-old *C.reinhardtii* cells were treated with 100 nM MC-LR for indicated days, with generation of H_2_S **(A)** and enzyme activity of DES **(B)** then measured. Cells without MC-LR treatment were used as the control. Values are means ± SD of three biological replicates.

Accumulated evidence demonstrates that H_2_S acts as a novel signal to modulate plant defense responses (Li et al., 2016; Corpas et al., 2019; Mei et al., 2019). O-acetylserine(thiol)lyase (OASTL) and D-Cys DES are the main enzymes responsible for H_2_S generation in plants (Alvarez et al., 2012; Mei et al., 2019). Based on our proteomics data, protein abundance of putative O-acetylserine(thiol)lyase (gi|5721691, gi|5726998) and DES (gi|5726568) increased after 1 d of MC-LR treatment and then decreased after 3 and 5 d of MC-LR treatment (**Table 1**). Because both of these proteins are associated with H_2_S biosynthesis (Jin et al., 2017), we explored the possible role of H_2_S signaling in *C. reinhardtii* in response to MC-LR treatment. We first determined the effects of MC-LR treatment on the production of H_2_S and activity of DES, which is responsible for H_2_S *in vivo* (Jin et al., 2017). As shown in **Figure 4A**, MC-LR treatment induced the rapid production of H_2_S, which peaked after 12 h of treatment and then quickly declined. For the *C. reinhardtii* control without MC-LR treatment, the content of H_2_S did not present an obvious change. In agreement with the generation profile of H_2_S, MC-LR treatment also increased the activity of DES, which reached a maximum level after 12 h of treatment and then declined after prolonged treatment (**Figure 4B**). Thus, these results indicate that MC-LR treatment increases DES activity for H_2_S generation during the early stage of exposure.

### H_2_S Protected *C. reinhardtii* From Toxicity of MC-LR by Scavenging ROS

**Figure 5:**
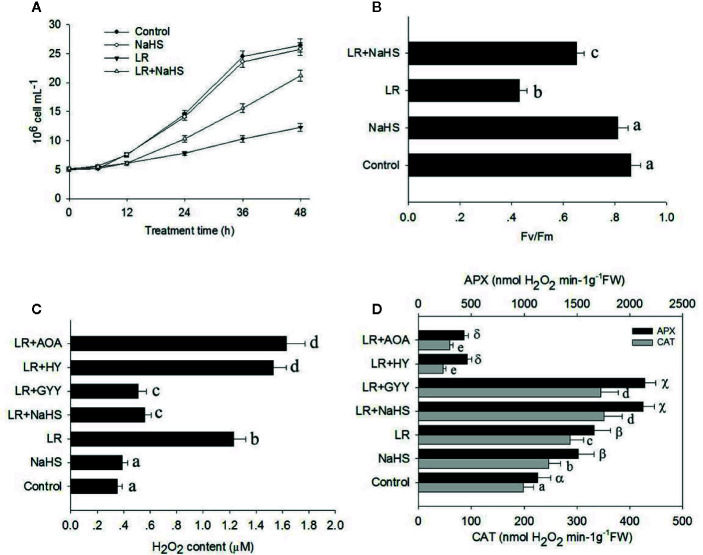
Effects of different H_2_S donors or H_2_S biosynthesis inhibitors on cell viability, H_2_O_2_ generation, and antioxidant enzyme activity. **(A, B)** Effects of MC-LR and H_2_S donor NaHS on cell density and photosynthesis Fv/Fm ratio of *C. reinhardtii*. Here, 3-d-old *C. reinhardtii* cells were treated with 100 nM MC-LR with or without H_2_S artificial donor NaHS (100 μM) for indicated days, with cell density **(A)** and photosynthesis Fv/Fm ratio **(B)** then calculated.Cells without MC-LR treatment were used as the control. Values are means ± SD of three biological replicates. **(C, D)** Effects of H_2_S biosynthesis scavenger or inhibitor on MC-LR-induced H_2_O_2_ generation and antioxidant APX and CAT activities. Here, 3-d-old *C. reinhardtii* cells were treated with 100 nM MC-LR with or without H_2_S artificial donor NaHS (100 μM), GYY4137 (GYY, 10 μM), H_2_S scavenger hypotaurine (HY, 10 μM), or H_2_S biosynthesis enzyme inhibitor aminooxyacetic acid (AOA, 1 mM) for 24 h, with H_2_O_2_ generation **(C)** and antioxidant APX and CAT activities **(D)** then measured. Values are means ± SD of three biological replicates.

To determine the function of H_2_S in *C. reinhardtii* responses to the MC toxin, we treated *C. reinhardtii* with the H_2_S donor NaHS and then compared cell density. As shown in **Figure 5A**, adding 10 µM NaHS alone did not obviously affect the growth of *C. reinhardtii*, whereas MC-LR treatment suppressed the growth of *C. reinhardtii*. However, NaHS treatment remarkably attenuated the inhibitory effect of MC-LR on the growth of *C. reinhardtii.* Additional NaHS treatment with MC-LR also increased the Fv/Fm ratio compared with that following MC-LR treatment alone (**Figure 5B**). These findings suggest that the H_2_S signaling enhances *C. reinhardtii* tolerance to MC toxicity.

As shown above, MC-LR induced the over-accumulation of ROS in *C. reinhardtii*. As H_2_S signaling is reported to scavenge ROS (Qi et al., 2019), we reasoned that additional H_2_S may reduce the accumulation of ROS in *C. reinhardtii.* Therefore, we treated *C. reinhardtii* with 100 nM MC-LR with or without H_2_S artifical donor NaHS, GYY, H_2_S scavenger hypotaurine (HY), H_2_S biosynthesis enzyme inhibitor aminooxyacetic acid (AOA) for 24 h, and then compared H_2_O_2_ accumulation. As shown in **Figure 5C**, the addition NaHS and GYY suppressed MC-LR-mediated H_2_O_2_ accumulation, whereas addition of AOA and HY aggravated MC-LR-induced H_2_O_2_ accumulation. Furthermore, we measured the activity of APX and CAT after MC-LR and different chemical treatment. As shown in **Figure 5D**, the addition of NaHS and GYY enhanced APX and CAT activity, but additional AOA and HY suppressed APX and CAT activity. These results suggest that H_2_S signaling protects *C. reinhardtii* from the damage induced by MC-LR through the up-regulation of APX and CAT activity to reduce H_2_O_2_ over-accumulation.

### H_2_S Signaling Suppressed MC-Induced Cell Autophagy

**Figure 6:**
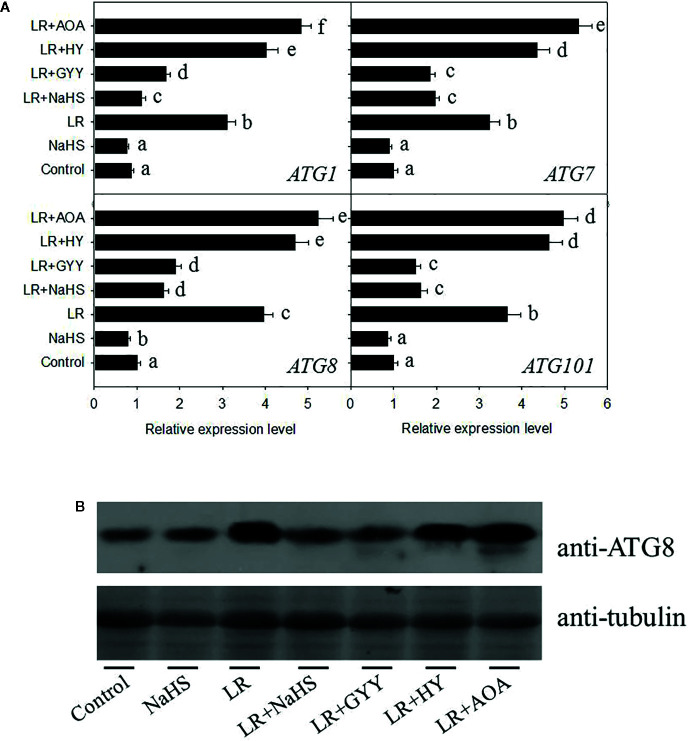
Effects of different H_2_S donors or H_2_S biosynthesis inhibitors on cell autophagy capability of *C. reinhardtii* under MC-LR treatment. **(A)** Effects of H_2_S biosynthesis scavenger or inhibitor on MC-LR-induced expression of *ATG1*, *AGT7*, *AGT8*, and *ATG101*. Here, 3-d-old *C. reinhardtii* cells were treated with 100 nM MC-LR with or without H_2_S artificial donor NaHS (100 μM), GYY4137 (GYY, 10 μM), H_2_S scavenger hypotaurine (HY, 10 μM), or H_2_S biosynthesis enzyme inhibitor aminooxyacetic acid (AOA, 1 mM) for 12 h, with transcriptional levels of *ATG1*, *AGT7*, *AGT8*, and *ATG101* then measured by qRT-PCR analysis. Values are means ± SD of three biological replicates. **(B)** Effects of H_2_S biosynthesis scavenger or inhibitor on MC-LR-induced protein accumulation of ATG8. Here, 3-d-old *C. reinhardtii* cells were treated with 100 nM MC-LR with or without H_2_S artificial donor for 3 d, with protein accumulation of ATG8 measured by Western blotting using anti-ATG8 antibody. Anti-Tubulin was used as the loading control.

Previous study has demonstrated that ROS induce cell autophagy, whereas H_2_S signaling suppresses cell autophagy (Alvarez et al., 2012), suggesting that cell autophagy may be involved in the toxic effects of MCs in *C. reinhardtii*. To check the effect of MC-LR on cell autophagy, we performed the qRT-PCR analysis to measure the transcriptional levels of *ATG1*, *AGT7*, *AGT8*, and *ATG101*, all of them are used as the autophagy-related marker genes. As shown in **Figure 6A**, we found that MC-LR treatment for 24 h markedly activated their expression, such activation effect could be attenuated by addition of H_2_S donors NaHS and GYY. Addition of H_2_S signal inhibitors AOA and HY further enhanced MC-LR-induced expressions of *ATG1*, *AGT7*, *AGT8*, and *ATG101*. These results suggest that MC-LR suppressed the growth of *C. reinhardtii* by activating the cell autophagy pathway. Consistently, we used the anti-ATG8 antibody to test the effects of H_2_S and MC-LR on the accumulation of ATG8, as a marker of cell autophagy. As shown in **Figure 6B**, MC-LR increased the protein abundance of ATG8, addition of NaHS and GYY repressed MC-LR-induced accumulation of ATG8, and addition of AOA and HY increased protein abundance of ATG8. These results support that MC-LR induces *C. reinhardtii* cell death through activation of the cell autophagy pathway.

## Discussion

As a by-product of bloom-forming cyanobacteria, MCs show toxicity to most aquatic microorganisms (Duan et al., 2009; Xu et al., 2018). Currently, however, the underlying mechanism remains unknown. In this study, we used unicellular alga *C. reinhardtii* to test the toxic effects of MC-LR. We found that MC-LR markedly suppressed *C. reinhardtii* growth, as presented by lower cell density and Fv/Fm ratio and higher cell death, suggesting general toxic allelopathic effects of MC-LR on *C. reinhardtii*. To better understand the detailed mechanism by which MC-LR attacks *C. reinhardtii*, we performed proteomic iTRAQ analysis and found several proteins that were differentially regulated by MC-LR treatment, including several defense proteins and antioxidant enzymes, suggesting that these proteins are possibly related to the effects of MC-LR on *C. reinhardtii* growth.

Among the differentially regulated proteins, the abundance of putative O-acetylserine(thiol)lyase and cysteine synthase increased at the beginning stage of MC-LR treatment. These proteins are both associated with the biosynthesis of H_2_S *in vivo* (Alvarez et al., 2012). In addition, H_2_S signaling is reported to enhance plant tolerance to environmental stress (Li et al., 2016), suggesting the possible role of H_2_S in the *C. reinhardtii* response to MC toxicity. Our data revealed that MC-LR indeed induced the rapid generation of H_2_S, accompanied by an increase in DES activity. Furthermore, we found that the addition of H_2_S donors NaHS and GYY enhanced the tolerance of *C. reinhardtii* to MC-LR toxicity by increasing the cell density and Fv/Fm ratio. In contrast, suppressing H_2_S biosynthesis by AOA and HY aggravated the toxicity of MCs on *C. reinhardtii* growth. These results suggest a novel function of H_2_S in protecting *C. reinhardtii* from MC toxicity.

ROS, mainly H_2_O_2_, are strongly induced by environmental stress to damage plants. As a result, plants have evolved a defense mechanism to avoid ROS-induced damage, e.g., enhancing antioxidant enzyme activity to scavenge ROS accumulation (Noctor et al., 2018; Smirnoff and Arnaud, 2019). Our proteomic data showed that MC-LR treatment up-regulated the protein abundance of APX and CAT at the beginning stage, suggesting that H_2_O_2_ signaling may be involved in *C. reinhardtii* responses to MC. We measured the dynamic changes in H_2_O_2_ and found that MC-LR treatment dramatically induced the accumulation of H_2_O_2_, which may be the main reason for the effects of MC on *C. reinhardtii* cell viability. Consistently, we analyzed the activity of APX and CAT. Although MC-LR induced increases in APX and CAT enzyme activity at the beginning stage, their activities decreased after long-term (e.g., 3 d) MC-LR treatment. It is possible that the reason why MC-LR caused an increase in ROS is because MC-LR did not efficiently sustain high levels of enzyme activity to successfully scavenge the over-accumulation of ROS. We demonstrated that H_2_S signaling activated antioxidant enzyme activity, thus it is possible that H_2_S enhanced the tolerance to *C. reinhardtii* against MC by activating antioxidant enzymes to scavenge ROS toxicity. We treated *C. reinhardtii* with MC-LR and additional H_2_S donors and found that the addition of NaHS and GYY improved cell viability, including an increase in cell density and Fv/Fm ratio. In contrast, inhibiting H_2_S biosynthesis with AOA and HY aggravated MC-LR toxicity on *C. reinhardtii* cell viability. Thus, these findings confirm the protective function of H_2_S against MC damage. We also found that the addition of H_2_S donors reactivated APX and CAT activity compared to that after MC-LR treatment alone, whereas addition of H_2_S biosynthesis inhibitors further suppressed enzyme activity. Therefore, we propose that H_2_S enhances the tolerance of *C. reinhardtii* cells to MC exposure by up-regulating and sustaining high levels of antioxidant enzyme activity to scavenge MC-LR-induced ROS.

ROS can induce cell autophagy (Huang et al., 2019; Signorelli et al., 2019; Tran et al., 2019). Here, we also found that MC-LR activated cell autophagy by increasing the transcriptional level of autophagy-related genes and protein abundance of ATG8. Thus, it is possible that MC-LR suppressed *C. reinhardtii* cell viability through ROS-induced cell autophagy. As H_2_S signaling negatively regulates cell autophagy (Wang et al., 2017), we also investigated the function of H_2_S in MC-LR-induced autophagy. We found that H_2_S suppressed cell autophagy because H_2_S donors suppressed MC-LR-induced expression of autophagy-related genes and ATG8 protein accumulation, whereas H_2_S biosynthesis inhibitors aggravated MC-LR-induced autophagy. We therefore propose that H_2_S-inhibited cell autophagy may also explain the protective role of H_2_S against MC damage.

**Figure 7:**
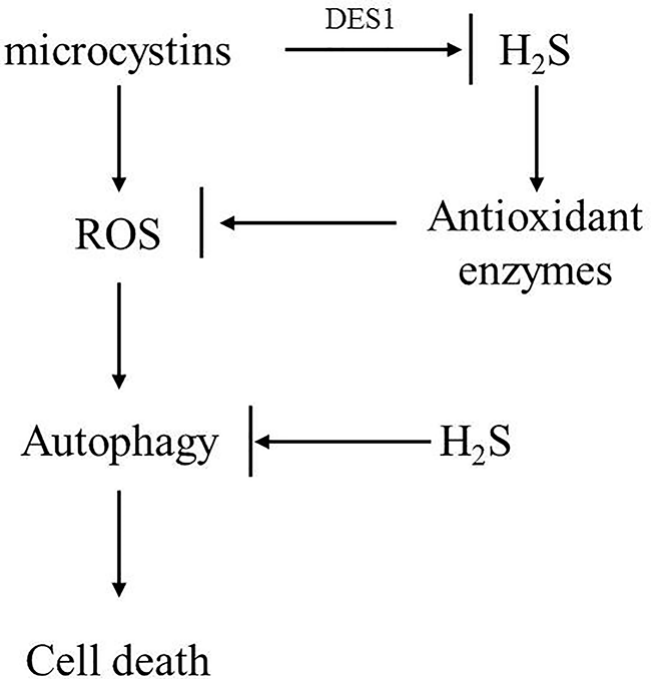
Possible model to illustrate novel role of H_2_S in mediating *C. reinhardtii* tolerance to toxic MC-LR treatment. In this model, we propose that MC-LR induced rapid accumulation of H_2_O_2_ and triggered cell autophagy to suppress cell growth and viability of *C. reinhardtii.* However, H_2_S acted as a novel signal to enhance tolerance of *C. reinhardtii* to MC-LR by potentiating antioxidant enzyme activity and suppressing cell autophagy.

In summary, we identified a novel function of H_2_S in enhancing *C. reinhardtii* cell tolerance to MC toxicity and proposed a model to explain the underlying mechanism. As shown in **Figure 7**, we propose that MC-LR treatment induced the strong accumulation of ROS, which subsequently activated cell autophagy to impair *C. reinhardtii* cell viability. Though MC-LR induced the accumulation of H_2_S and increased APX and CAT antioxidant enzyme activities to reduce the accumulation of ROS, this effect was inadequate and only occurred at the beginning stage, resulting in the over-accumulation of ROS and damage to *C. reinhardtii* cell viability. However, additional H_2_S could sustain the high level of antioxidant enzyme activity, as well as suppress cell autophagy, ultimately enhancing the tolerance of *C. reinhardtii* to MC toxicity. Taken together, our findings reveal a novel mechanism by which H_2_S acts as a signal to improve *C. reinhardtii* tolerance to MC by activating antioxidant enzyme activity and suppressing cell autophagy.

## Data Availability Statement

The raw data supporting the conclusions of this article will be made available by the authors, without undue reservation.

## Author Contributions

X-DC, X-YH, and A-QJ designed the research. X-DC and YL performed the experiments. X-DC, L-MY, X-YH, and A-QJ analyzed the data. X-DC, X-YH, and A-QJ wrote the paper.

## Funding

This study was funded by the National Key Research and Development Program of China (2017YFD0201401), National Natural Science Foundation of China (41766006), and Six Talent Peaks Project in Jiangsu Province.

## Conflict of Interest

The authors declare that the research was conducted in the absence of any commercial or financial relationships that could be construed as a potential conflict of interest.

## Supplementary Material

Supplemental Figure 1 Dose effect of MC-LR on growth **(A)** and cell death **(B)** of *C. reinhardtii*. Here, 3-d-old *C. reinhardtii* cells were treated with MC-LR at different concentrations for 72 h, with cell density **(A)** and cell death **(B)** then measured. Values are means ± SD of three biological replicates.

Supplemental Figure 2 Dose effect of MC-LR on cell photosynthesis capability of *C. reinhardtii*. Here, 3-d-old *C. reinhardtii* cells were treated with MC-LR at different concentrations for 6 d, with Fv/Fm ratio then calculated. Values are means ± SD of three biological replicates.
