# Supplementary figures and images for: Hydrogen Sulfide Signaling Protects Chlamydomonas reinhardtii Against Allelopathic Damage From Cyanobacterial Toxin Microcystin-LR
Source: Front Plant Sci. 2020 Jul 17;11:1105. doi: 10.3389/fpls.2020.01105 (PMC7379851; doi:10.3389/fpls.2020.01105)

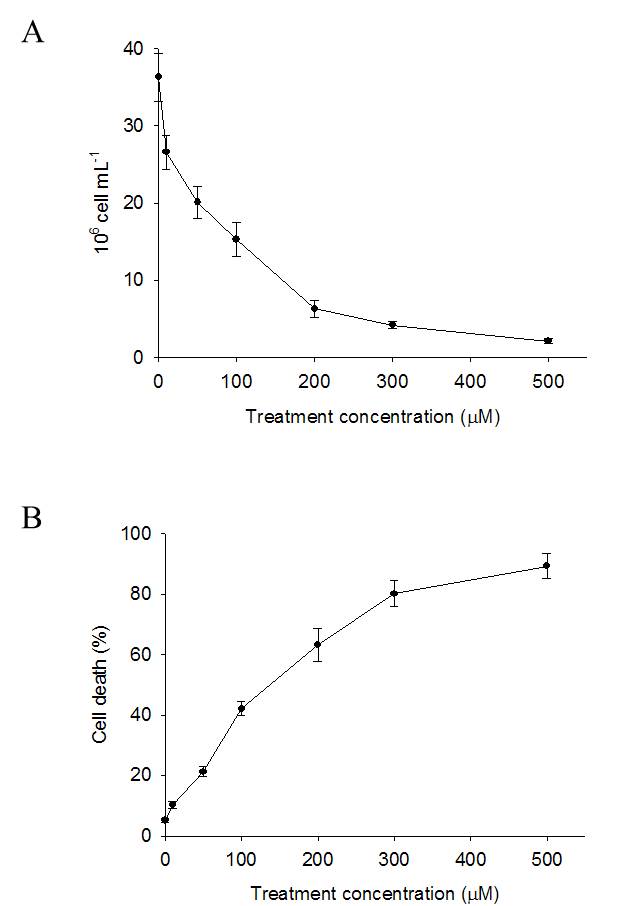

Supplement: Supplemental Figure 1 — Dose effect of MC-LR on growth (A) and cell death (B) of C. reinhardtii. Here, 3-d-old C. reinhardtii cells were treated with MC-LR at different concentrations for 72 h, with cell density (A) and cell death (B) then measured. Values are means ± SD of three biological replicates. [file Image_1.jpeg]

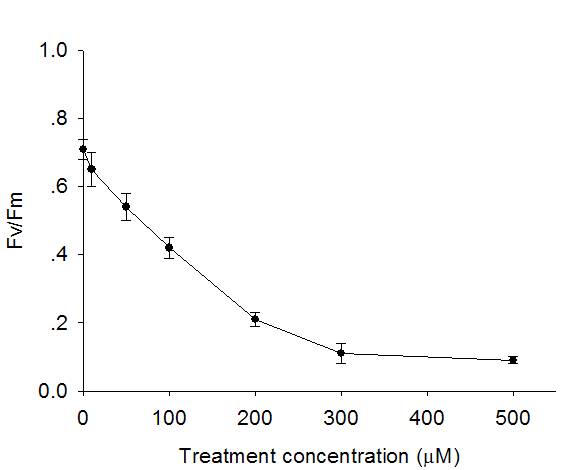

Supplement: Supplemental Figure 2 — Dose effect of MC-LR on cell photosynthesis capability of C. reinhardtii. Here, 3-d-old C. reinhardtii cells were treated with MC-LR at different concentrations for 6 d, with Fv/Fm ratio then calculated. Values are means ± SD of three biological replicates. [file Image_2.jpeg]
