# Supplementary material for: Hydrogen Sulfide Signaling Protects Chlamydomonas reinhardtii Against Allelopathic Damage From Cyanobacterial Toxin Microcystin-LR
Source: Front Plant Sci. 2020 Jul 17;11:1105. doi: 10.3389/fpls.2020.01105 (PMC7379851; doi:10.3389/fpls.2020.01105)
Supplement: Supplementary file 3 [file Table_1.docx]

| QRT-PCR primer for Tubulin | |
| --- | --- |
| Forward primer sequence | AGTGGATTCCCAACAACGTC |
| Reverse primer sequence | GACTCGGCCTCAGTGAACTC |
| QPT-PCR for ATG1 | |
| Forward primer sequence | GGGTGCGGTGGTGTACTAGC |
| Reverse primer sequence | AGTCCTCTTCCGGCACCGAT |
| QPT-PCR for ATG7 | |
| Forward primer sequence | GCATTGTAGGAGGGTGGTAGGAG |
| Reverse primer sequence | CTCCAGGTCTGTGTGGCTAGCTC |
| QPT-PCR for ATG8 | |
| Forward primer sequence | CAGCATCTCCACAATGGTTGGC |
| Reverse primer sequence | CTCTGCCTTCTCGACAATGACTGG |
| QPT-PCR for ATG101 | |
| Forward primer sequence | CTTCTGTGCCCAGGTTGACAAGC |
| Reverse primer sequence | GCTCAACACCCACGTTTCCCAG |

Supplemental Table 1: Primer sequences used in this study
